# View-Aligned Nonlocal Low-Rank Tensor Reconstruction for Snapshot Compressive Multi-View Spectral Imaging System

**DOI:** 10.3390/s26154875

**Published:** 2026-08-02

**Authors:** Xiaorui Yin, Lijuan Su, Yu Wang, Yan Yuan

**Affiliations:** Key Laboratory of Precision Opto-Mechatronics Technology, Ministry of Education, Beihang University, Beijing 100191, China; xryin@buaa.edu.cn (X.Y.); wangyu_2025@buaa.edu.cn (Y.W.); yuanyan@buaa.edu.cn (Y.Y.)

**Keywords:** computational optical imaging, snapshot compressive imaging, multi-view spectral imaging, coded optical sensing, view alignment, nonlocal low-rank tensor reconstruction, ADMM

## Abstract

Snapshot compressive multi-view spectral imaging (SC-MVSI) multiplexes view-spectral information into a single coded measurement, enabling compact acquisition with a two-dimensional detector. Because each reconstructed channel corresponds to both a selected spectral response and a view direction, direct cross-channel modeling at identical pixel coordinates can introduce structural mismatch caused by view-dependent displacement. This paper proposes a reference-guided view-aligned nonlocal low-rank tensor reconstruction method for SC-MVSI. The reconstruction is formulated as a coded inverse problem and solved using the alternating direction method of multipliers (ADMM) in a variable-splitting framework. In the prior update, a reference tensor guides block-level patch alignment before nonlocal tensor grouping, and the resulting fourth-order tensor groups are regularized by canonical polyadic (CP) low-rank approximation. Experiments on eight synthesized multispectral light-field scenes show that the proposed method achieves the highest average PSNR of 33.61 dB and the lowest average CAE of 5.69 degrees among the compared baselines, while obtaining the second-highest average SSIM of 0.8823. Real-system experiments further provide a qualitative demonstration of applying the proposed reconstruction framework to captured coded measurements.

## 1. Introduction

Multi-view spectral imaging plays an important role in optical imaging systems because it records spectral responses together with observations from multiple view directions. Multispectral filter-array imaging architectures and compressive hyperspectral reconstruction studies show that spectral imaging increasingly couples optical encoding, detector sampling, and computational recovery [1,2,3]. Multispectral light-field imaging further combines angular sampling with spectral selectivity. Studies on light-field imaging and integral imaging show that multi-view measurements retain angular diversity and view-dependent parallax, which may provide geometric cues for downstream analysis of scene structure or depth estimation when combined with an explicit geometric model [4,5]. These angular cues can also support occlusion-aware observation and occlusion removal [6]. In parallel, multispectral snapshot and spatial–spectral sensing studies have shown that joint spatial and spectral acquisition is useful for material-dependent interpretation and classification [7,8]. Such integration is attractive for dynamic targets and compact imaging platforms, where snapshot spectral and spectral light-field imaging can reduce scanning requirements and improve temporal sampling [2,9]. However, conventional scanning or sequential spectral imaging systems remain less suitable for dynamic imaging because time-consuming acquisition can introduce temporal delays and registration difficulties [2].

Snapshot compressive imaging (SCI) provides an effective route for acquiring high-dimensional information with a two-dimensional (2D) detector. By optically multiplexing multiple dimensions before detection, coded snapshot systems can record compressed measurements of spectral information [2,3,10], angular information [11], temporal or spatiotemporal information [10,12], or geometry-related information [11], and then recover the desired signal computationally. Recent optical and algorithmic co-design studies have shown that coded modulation and reconstruction models can make compressive hyperspectral imaging more practical for real imaging scenarios [13,14,15,16]. Related coded light-field and multispectral light-field imaging studies further indicate that angular and spectral information can be jointly captured or jointly processed in compact optical configurations [4,17,18]. Ultra-compact snapshot spectral light-field imaging has also been demonstrated as an effective optical acquisition strategy for spectral light-field information [9]. Complementary to such system-level acquisition studies, this paper focuses on reconstruction from snapshot compressive multi-view spectral imaging (SC-MVSI) measurements. The reconstruction target in SC-MVSI differs from a conventional single-view spectral cube because each channel is associated with both a view direction and a selected spectral response.

Existing reconstruction methods for snapshot compressive spectral imaging [3,10] can be broadly discussed as model-based methods [19,20,21], end-to-end deep learning-based methods [22,23,24,25], and deep unfolding-based methods [26,27,28]. Model-based methods combine the physical measurement model with handcrafted or image-adaptive priors, such as sparsity [29], total variation [30,31], nonlocal similarity [19], plug-and-play denoising [20,32], and low-rank constraints [21,33,34]. End-to-end deep learning-based methods directly learn the mapping from coded measurements to reconstructed spectral images or spectral cubes, while deep unfolding-based methods embed iterative optimization steps into trainable network architectures. Self-supervised, zero-shot, and untrained-network strategies can reduce the dependence on paired training data by adapting image-specific priors directly from coded observations [35,36,37,38,39]. Although end-to-end and deep unfolding methods have substantially improved reconstruction accuracy and efficiency, their direct application to SC-MVSI is limited by the lack of sufficiently large and representative multi-view spectral datasets and by the need for training data matched to the sensing matrix, coding pattern, channel configuration, and compression ratio.

SC-MVSI records multiple view-spectral channels in a single exposure, each corresponding to a specific viewing direction and spectral response. Because the viewing directions are fixed by the optical configuration, corresponding scene structures may appear at different pixel locations across channels. These reconstruction methods generally exploit cross-channel correlation without explicitly modeling this geometric correspondence. As a result, patches extracted at the same pixel coordinates may represent different scene points, weakening cross-channel low-rank modeling, especially around object boundaries, occlusions, and textured regions.

To address this problem, this paper proposes a view-aligned nonlocal low-rank tensor reconstruction method for SC-MVSI. The key idea is to construct the low-rank prior on geometrically aligned view-spectral patches rather than on spatially co-located cross-channel patches. Specifically, a reference tensor is used to estimate block-level channel offsets, and local patches from different view-spectral channels are aligned before tensor grouping. The view-aligned patch tensors are then grouped with nonlocal similar tensors to form a fourth-order tensor that jointly represents local spatial structure, cross-view spectral correlation, and nonlocal self-similarity. A canonical polyadic (CP) low-rank approximation is introduced to regularize these fourth-order tensor groups [33,34,40].

To make this view-aligned prior compatible with the coded measurement model, it is embedded into a snapshot compressive reconstruction framework and solved with the alternating direction method of multipliers (ADMM) [41]. The resulting optimization process is decomposed into a data-consistency subproblem and a nonlocal tensor low-rank regularization subproblem. During the iterative reconstruction, a reference tensor is introduced to provide a stable structural guide for block-level offset estimation, reducing the influence of early-stage artifacts on patch alignment. Through reference-guided view-aligned tensor construction, nonlocal grouping, CP low-rank approximation, and overlapping patch aggregation, the proposed method aims to recover multi-channel images while preserving both geometric correspondence and channel-response structure.

The main contributions of this work are summarized as follows:

- A reference-guided view-aligned nonlocal tensor construction strategy is designed for the view-spectral compound channels of SC-MVSI. By estimating block-level offsets from a reference tensor and aligning local patches across view channels before nonlocal grouping, this strategy mitigates the spatial mismatch caused by direct cross-channel patch stacking.
- An ADMM-based reconstruction framework is developed to couple the coded measurement model with the proposed view-aligned tensor prior. The data-consistency update enforces agreement with the detector measurement, while the prior update regularizes geometrically aligned view-spectral patch groups through nonlocal CP low-rank approximation.
- The effectiveness of the proposed method is evaluated using synthesized multispectral light-field scenes, real-system imaging experiments, and ablation studies. Quantitative and visual comparisons with representative snapshot compressive reconstruction algorithms are provided, and the roles of reference-guided alignment and nonlocal tensor grouping are analyzed.

The remainder of this paper is organized as follows. Section 2 describes the SC-MVSI system and imaging model, including the optical acquisition principle, coded measurement formation, and view-spectral displacement model. Section 3 presents the view-aligned nonlocal low-rank tensor reconstruction method. Section 4 reports the experimental settings and results. Section 5 discusses the findings and limitations. Section 6 concludes this work.

## 2. SC-MVSI System and Imaging Model

This section describes the physical and mathematical basis of the SC-MVSI system before introducing the reconstruction algorithm. The optical acquisition path is first summarized to clarify how view-spectral channels are separated, filtered, encoded, and aggregated on the detector. The coded measurement is then formulated as a modulation-and-summation model, followed by a block-level view-spectral displacement model that motivates the view-aligned tensor construction used in the proposed method.

**Figure 1:**
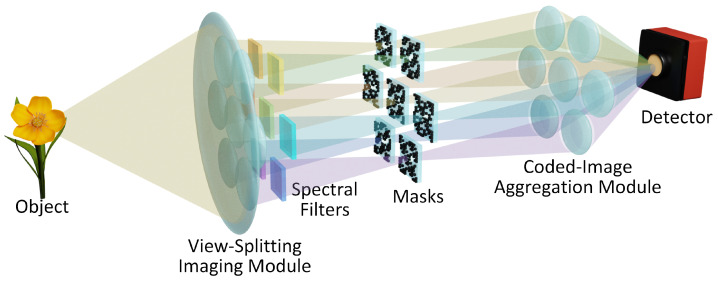
Optical acquisition principle of the snapshot compressive multi-view spectral imaging (SC-MVSI) system. The object is separated into multiple view-spectral channels by the view-splitting imaging module and spectral filters, modulated by masks, and then superimposed on the detector through the coded-image aggregation module.

The optical acquisition path is illustrated in Figure 1. The view-splitting imaging module first separates the object information into multiple view channels, and spectral filters assign different spectral responses to these channels. After filtering, each view-spectral channel is modulated by its corresponding mask. The coded channel images are then relayed and superimposed by the coded-image aggregation module onto the detector, producing a multiplexed coded measurement. The tensor and mask notation used to describe this process is introduced in the coded measurement model below.

### 2.1. Coded Measurement Formation

Let $X\in R^{H\times W\times K}$ denote the view-spectral channel tensor to be reconstructed, where *H* and *W* are the spatial dimensions and *K* is the number of encoded channels. The *k*th frontal slice $X^{\left(k\right)}\in R^{H\times W}$ corresponds to the *k*th view-spectral channel associated with a selected spectral channel centered at $\lambda_{k}$. Let $C\in R^{H\times W\times K}$ denote the coding-pattern tensor, whose *k*th frontal slice $C^{\left(k\right)}\in R^{H\times W}$ is the coding pattern for the *k*th channel. The 2D coded measurement is denoted as $Y\in R^{H\times W}$.

The coded acquisition process can be represented by a linear measurement operator A(·;C):

(1) Y=A(X;C)+N,

where $N\in R^{H\times W}$ denotes measurement noise. In the snapshot compressive measurement model, A(·;C) modulates each channel with its corresponding coding pattern and sums the modulated channels on the detector:

(2) $$A\left(X;C\right)=\sum_{k=1}^{K}C^{\left(k\right)}⊙X^{\left(k\right)},$$

where C is fixed during reconstruction, $C^{\left(k\right)}$ is its *k*th coding slice, and ⊙ denotes the Hadamard product. For simplicity, A(X) is used in later sections when the coding tensor is fixed. This formulation is used only to describe the physical measurement consistency of SC-MVSI. The key difference from conventional coded aperture snapshot spectral imaging (CASSI) is not the linear compression form itself, but the view-spectral meaning of the channel dimension.

**Figure 2:**
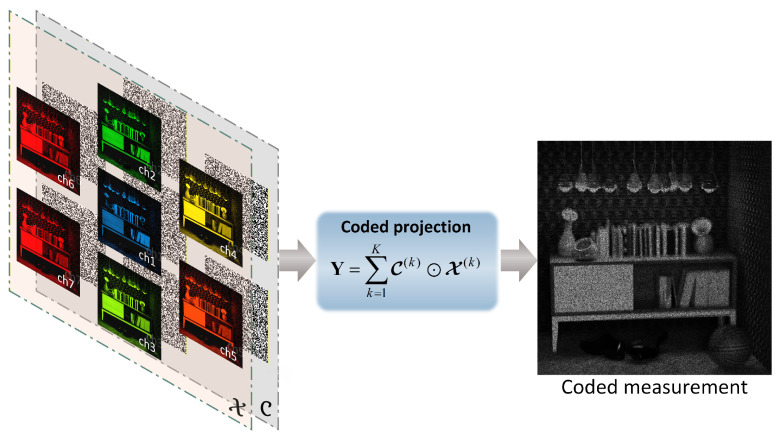
Coded measurement formation in the SC-MVSI system. Each view-spectral channel slice $X^{\left(k\right)}$ is modulated by its corresponding binary mask $C^{\left(k\right)}$, and all modulated slices are summed on the detector to form Y.

Figure 2 visualizes this modulation-and-summation process. In the current SC-MVSI prototype and simulation setting, one coded measurement records K=7 view-spectral channels. Each view-spectral channel slice is modulated by its corresponding binary mask, and all modulated slices are integrated into one 2D coded measurement.

### 2.2. View-Spectral Displacement Model

In conventional CASSI reconstruction, different channel slices are usually regarded as spectral bands observed from the same viewpoint. In SC-MVSI, however, the channel dimension jointly encodes view direction and spectral response. Therefore, for the same scene point, its projection in different channel slices may appear at different spatial positions due to view-dependent displacement.

Let $p_{i}$ be the spatial location of the *i*th reference image block, and let $b_{k}\in R^{2}$ be the unit baseline direction from the reference channel to the *k*th channel, with $b_{1}=0$ for the center reference channel. In the implemented reconstruction, the cross-view displacement is represented as one scalar offset for each image block rather than as a dense pixel-wise field. The corresponding block location in the *k*th channel can be written as

(3) $$p_{i,k}=p_{i}+d_{i}b_{k},$$

where $d_{i}$ is the scalar offset assigned to the *i*th block. Equivalently, the channel-dependent displacement vector is $d_{i,k}=d_{i}b_{k}$. This relation implies that image patches extracted from identical pixel coordinates across channels do not necessarily describe the same physical scene structure.

**Figure 3:**
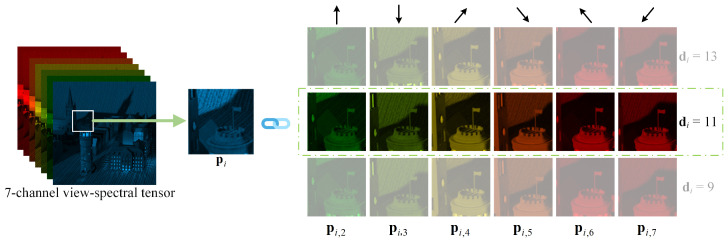
Block-level view-spectral displacement model. A reference block at $p_{i}$ is matched across the view-spectral channels by searching scalar offsets $d_{i}$. For each candidate offset, the extraction locations satisfy $p_{i,k}=p_{i}+d_{i}b_{k}$. The arrows indicate displacement directions $b_{k}$, and the dashed box highlights the patches at the selected offset $d_{i}$.

Figure 3 illustrates the block-level displacement model. Starting from a reference block at $p_{i}$, different candidate offsets $d_{i}$ shift the extraction locations in the remaining view-spectral channels along the corresponding baseline directions. The selected offset determines the aligned block locations ${\left\{p_{i,k}\right\}}_{k=1}^{K}$ used for subsequent tensor construction.

If a cross-channel tensor group is directly formed from co-located patches, local structures from different scene points may be stacked into the same tensor. This issue becomes more evident around object boundaries, occlusion regions, and areas with large view-dependent shifts. Consequently, the low-rankness of the tensor group may be weakened not because the scene lacks multi-channel correlation, but because the tensor group is constructed without view alignment. In this work, block-level offsets are therefore considered in the construction of nonlocal tensor groups rather than introduced as additional global reconstruction variables.

## 3. View-Aligned Nonlocal Low-Rank Tensor Reconstruction

This section presents the proposed reconstruction method based on the SC-MVSI forward model. The reconstruction objective and ADMM variable splitting are first formulated to separate measurement consistency from prior regularization. The prior update is then detailed through reference-guided block-offset estimation, view-aligned nonlocal tensor grouping, CP low-rank approximation, overlapping aggregation, and the final algorithm summary.

### 3.1. Reconstruction Formulation and ADMM Framework

Based on the coded measurement model, SC-MVSI reconstruction is formulated as a regularized inverse problem:

(4) $$\min_{X}\frac{1}{2}{∥A(X)-Y∥}_{F}^{2}+\gamma R\left(X\right),$$

where R(·) denotes the view-aligned nonlocal low-rank tensor prior and γ is the regularization parameter. The first term is the measurement-consistency term. It constrains the reconstructed view-spectral tensor to reproduce the coded detector measurement after optical modulation and summation by A(·). Therefore, this term is determined by the SC-MVSI forward model and does not encode any assumption about scene structure.

The second term introduces prior knowledge about the reconstructed tensor. Unlike conventional spectral SCI, where adjacent channels are usually treated as spatially registered spectral bands, SC-MVSI contains view-dependent displacement in the channel dimension. Therefore, R(X) is not a co-located cross-channel smoothness or low-rank prior. It represents a view-aligned nonlocal tensor prior, in which block-level patch alignment is required before cross-channel patch stacking and nonlocal tensor grouping. In this way, the regularization acts on geometrically corresponding view-spectral structures rather than on patches extracted from identical pixel coordinates.

Equation (4) defines the reconstruction problem studied in this paper. The block-level offsets are not introduced as independent global unknowns in the objective; instead, they are estimated locally during tensor-prior construction. This keeps the physical reconstruction variable as X while allowing the prior term to account for view-dependent geometry.

**Figure 4:**
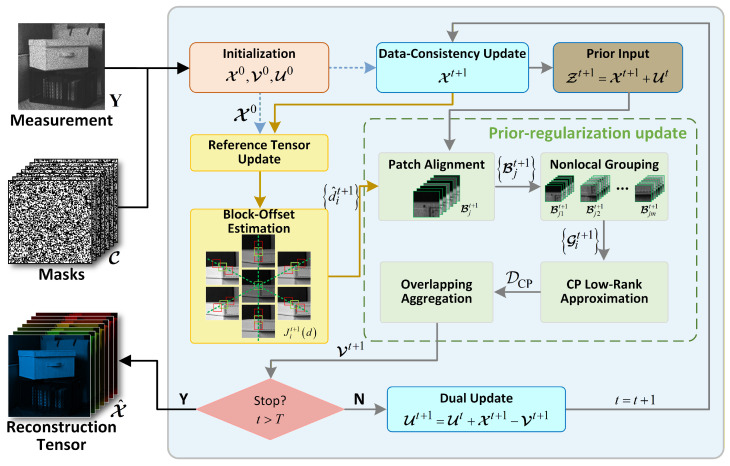
Overall workflow of the proposed reconstruction method. The framework alternates between data-consistency reconstruction and reference-guided prior regularization, where block-level view alignment supports patch grouping, canonical polyadic (CP) low-rank approximation, and overlapping aggregation.

The proposed reconstruction method solves Equation (4) by embedding a view-aligned nonlocal low-rank tensor prior into an ADMM reconstruction framework. As illustrated in Figure 4, the framework contains two coupled parts: a data-consistency update determined by the coded measurement model, and a prior-regularization update that constructs view-aligned nonlocal tensor groups from a reference tensor and regularizes them through CP low-rank approximation. This section first introduces the ADMM variable splitting and update equations, and then describes the reference-guided block-offset estimation, tensor grouping, low-rank approximation, and overlapping aggregation used in the prior update.

To decouple the measurement-consistency term and the nonlocal tensor prior in Equation (4), an auxiliary tensor $V\in R^{H\times W\times K}$ is introduced. The optimization problem is rewritten as

(5) $$\min_{V,X}\frac{1}{2}{∥A(X)-Y∥}_{F}^{2}+\gamma R\left(V\right),\;s.t.\;X=V.$$

Here, X is updated by the measurement-consistency subproblem, and V is its auxiliary copy updated by the prior-regularization subproblem. The equality constraint X=V is only an ADMM variable splitting strategy. The view alignment is implemented inside the nonlocal tensor construction used by R(V), which keeps the optimization consistent with the physical reconstruction variable and with the subsequent experimental implementation.

Using the scaled dual tensor U, the augmented Lagrangian of Equation (5) can be written as

(6) $$L_{\rho }\left(X,V,U\right)=\frac{1}{2}{∥A(X)-Y∥}_{F}^{2}+\gamma R\left(V\right)+\frac{\rho }{2}{∥X-V+U∥}_{F}^{2},$$

where ρ is the penalty parameter. The ADMM iterations contain three updates.

First, the measurement-consistency variable is updated by

(7) $$X^{t+1}=\arg\min_{X}\frac{1}{2}{∥A(X)-Y∥}_{F}^{2}+\frac{\rho }{2}{∥X-V^{t}+U^{t}∥}_{F}^{2}.$$

This subproblem restores the consistency between the reconstructed tensor and the coded measurement. Let $T^{t}=V^{t}-U^{t}$ denote the current ADMM prediction before measurement correction. For the coded modulation-and-summation operator, applying the Sherman–Morrison formula pixel-wise gives the following tensor-form closed-form update:

(8) $$X^{t+1}=T^{t}+A^{*}\left(\frac{Y-A\left(T^{t}\right)}{\rho 1+\sum_{k=1}^{K}C^{\left(k\right)}⊙C^{\left(k\right)}}\right),$$

where $A^{*}\left(\cdot \right)$ is the adjoint back-projection operator of A(·), $1\in R^{H\times W}$ is an all-one matrix, and the fraction denotes element-wise division. The correction term is obtained from the mismatch between the detector measurement and the coded projection of $T^{t}$. The denominator combines the ADMM penalty and the accumulated mask energy, written as the sum of the Hadamard product of each mask slice with itself; therefore, the expression applies to both binary and grayscale coding masks.

Second, the prior-regularization variable is updated by

(9) $$V^{t+1}=\arg\min_{V}\gamma R\left(V\right)+\frac{\rho }{2}{∥X^{t+1}-V+U^{t}∥}_{F}^{2}.$$

For clarity, the input of this prior update is denoted as

$$Z^{t+1}=X^{t+1}+U^{t}.$$

The subproblem is implemented by applying the reference-guided nonlocal tensor low-rank operator to $Z^{t+1}$. The following parts describe this prior update in detail.

### 3.2. Reference-Guided Block-Offset Estimation

The reference tensor $X_{\mathrm{ref}}^{t+1}\in R^{H\times W\times K}$ provides a stable structural guide for block-level offset search and nonlocal similar-patch grouping in the t+1 prior update. At early iterations, direct matching from the current reconstruction can be affected by coding artifacts. Therefore, the implementation uses a coarse reconstruction $X_{\mathrm{GAP}}\in R^{H\times W\times K}$, obtained with the GAP-TV solver, as the initial matching reference. Let $\tau_{\mathrm{init}}$ denote the number of initial ADMM iterations for which this reference is kept fixed. After this initialization interval, the current reconstruction estimate is directly used as the self-guided reference tensor. Thus, the reference tensor is updated as

(10) $$X_{\mathrm{ref}}^{0}=X_{\mathrm{GAP}},\;X_{\mathrm{ref}}^{t+1}=\left\{\begin{array}{cc} X_{\mathrm{GAP}},\; & t+1\leq \tau_{\mathrm{init}},\\ X^{t+1},\; & t+1>\tau_{\mathrm{init}}. \end{array}\right.$$

The block offsets are estimated from $X_{\mathrm{ref}}^{t+1}$ rather than from the noisier prior input $Z^{t+1}$, while the actual patch values used for reconstruction are still extracted from $Z^{t+1}$.

For the *i*th reference block, the method searches a scalar offset from a discrete candidate set D. Let $P_{i,k}^{t+1}\left(d\right)\in R^{s\times s}$ denote the candidate reference patch extracted from the *k*th channel of $X_{\mathrm{ref}}^{t+1}$ at location $p_{i}+db_{k}$, where *s* is the patch size. The matching cost of a candidate offset *d* is computed by comparing the center-view patch with all edge-view patches:

(11) $$J_{i}^{t+1}\left(d\right)=\frac{1}{\sum_{k=2}^{K}w_{k}}\sum_{k=2}^{K}w_{k}\phi \left(P_{i,1}^{t+1}\left(0\right),P_{i,k}^{t+1}\left(d\right)\right),$$

where $w_{k}$ is the channel weight and ϕ(·,·) is the patch matching cost. All off-axis channels are equally weighted in this implementation, with $w_{k}=1$ for k=2,…,K. In this implementation, ϕ(·,·) is computed using gradient-based zero-mean normalized cross-correlation, which compares local structural consistency rather than raw intensity differences. The selected raw offset is

(12) $${\tilde{d}}_{i}^{t+1}=\arg\min_{d\in D}J_{i}^{t+1}\left(d\right).$$

The estimated block offset ${\tilde{d}}_{i}^{t+1}$ is directly used for the subsequent view-aligned tensor construction.

### 3.3. View-Aligned Nonlocal Tensor Grouping

After the block offsets are obtained, the actual patch values are extracted from the prior input tensor $Z^{t+1}$. The aligned patch matrix $B_{i,k}^{t+1}\in R^{s\times s}$ extracted from the *k*th channel can be expressed as

(13) $$B_{i,k}^{t+1}=E_{k}\left(Z^{t+1},p_{i}+{\tilde{d}}_{i}^{t+1}b_{k}\right),$$

where $E_{k}\left(\cdot \right)$ denotes the patch extraction operator in the *k*th channel. Thus, the scalar block offset ${\tilde{d}}_{i}^{t+1}$ determines the channel-dependent extraction locations through the known baseline directions ${\left\{b_{k}\right\}}_{k=1}^{K}$. The aligned patches from all channels are stacked into a local view-spectral tensor. Similar local tensors are further searched within a nonlocal window Ω and stacked to form a fourth-order tensor group:

(14) $$B_{j}^{t+1}={\mathrm{stack}}_{k=1}^{K}\left(B_{j,k}^{t+1}\right)\in R^{s\times s\times K},$$

(15) $$G_{i}^{t+1}\left(:,:,:,m\right)=B_{j_{m}}^{t+1},\;j_{m}\in N_{i}^{t+1},\;m=1,\ldots ,M.$$

where $B_{j}^{t+1}$ denotes a single view-aligned local view-spectral tensor constructed at the *j*th candidate patch center, $N_{i}^{t+1}=\left\{j_{1},\ldots ,j_{M}\right\}$ is the set of *M* nonlocal candidates selected for the *i*th reference patch, and $G_{i}^{t+1}$ is obtained by concatenating these local tensors along the fourth dimension. Equivalently, this reference-guided grouping process can be written compactly as

(16) $$G_{i}^{t+1}=Q_{i}\left(Z^{t+1};X_{\mathrm{ref}}^{t+1}\right)\in R^{s\times s\times K\times M},$$

where $Q_{i}\left(\cdot \right)$ denotes the reference-guided grouping operator that includes view-aligned patch extraction, nonlocal candidate selection, and fourth-mode stacking. The group $G_{i}^{t+1}$ jointly represents local spatial structure, view-spectral correlation, and nonlocal self-similarity.

### 3.4. CP Low-Rank Approximation and Overlapping Aggregation

For each tensor group $G_{i}^{t+1}$, a CP low-rank approximation is used to suppress noise and coding artifacts while preserving multi-dimensional correlations. The approximation can be written as

(17) $$G_{i}^{t+1}\approx \sum_{r=1}^{R_{i}}a_{r}^{\left(1\right)}\circ a_{r}^{\left(2\right)}\circ a_{r}^{\left(3\right)}\circ a_{r}^{\left(4\right)},$$

where $R_{i}$ is the CP rank of the *i*th tensor group, $a_{r}^{\left(n\right)}$ denotes the factor vector in the *n*th mode, and ∘ denotes the vector outer product. After low-rank approximation, the denoised tensor groups are returned to their aligned spatial positions and averaged over overlaps:

(18) $$V^{t+1}=M_{\mathrm{agg}}\left({\left\{D_{\mathrm{CP}}\left(G_{i}^{t+1}\right)\right\}}_{i}\right),$$

where $D_{\mathrm{CP}}\left(\cdot \right)$ denotes CP low-rank approximation and $M_{\mathrm{agg}}\left(\cdot \right)$ denotes normalized overlapping patch aggregation. In the ADMM framework, this operation serves as the practical proximal update associated with the nonlocal low-rank tensor prior in Equation (9).

After obtaining $X^{t+1}$ and $V^{t+1}$, the scaled dual tensor is updated as

(19) $$U^{t+1}=U^{t}+X^{t+1}-V^{t+1}.$$

### 3.5. Algorithm Summary

Algorithm 1 summarizes the ADMM reconstruction loop and the reference-guided prior update shown in Figure 4.
**Algorithm 1** Proposed view-aligned nonlocal low-rank tensor reconstruction method   **Input:** Y, C, A; parameters $\rho ,s,\Omega ,M,D,{\left\{b_{k}\right\}}_{k=1}^{K},\tau_{\mathrm{init}},T,\varepsilon$.   **Output:** Reconstructed view-spectral tensor $\hat{X}$.  1:Obtain $X_{\mathrm{GAP}}$ and initialize $X^{0}=X_{\mathrm{GAP}}$, $V^{0}=X^{0}$, $U^{0}$ as a zero tensor, and $X_{\mathrm{ref}}^{0}=X_{\mathrm{GAP}}$.  2:**for** t=0,1,…,T−1 **do**  3:    Update $X^{t+1}$ by the data-consistency solution in Equation (8).  4:    Form the prior input $Z^{t+1}=X^{t+1}+U^{t}$.  5:    Update the reference tensor $X_{\mathrm{ref}}^{t+1}$ using Equation (10).  6:    **for** each reference patch center $p_{i}$ **do**  7:        Estimate ${\tilde{d}}_{i}^{t+1}$ by minimizing $J_{i}^{t+1}\left(d\right)$ over D using Equations (11) and (12).  8:        Construct the view-aligned nonlocal tensor group $G_{i}^{t+1}=Q_{i}\left(Z^{t+1};X_{\mathrm{ref}}^{t+1}\right)$.  9:        Compute the CP low-rank approximation $D_{\mathrm{CP}}\left(G_{i}^{t+1}\right)$.10:    **end for**11:    Aggregate all restored groups to obtain $V^{t+1}$ using Equation (18).12:    Update $U^{t+1}$ using Equation (19).13:    **if** $∥X^{t+1}-X^{t}{∥}_{F}/\max(∥X^{t}{∥}_{F},\varepsilon )<\varepsilon$ **then**14:        **break**15:    **end if**16:**end for**17:Set $\hat{X}=X^{t+1}$.

## 4. Experiments and Results

This section evaluates the proposed method using synthesized multispectral light-field data, ablation experiments, and real-system measurements. The experimental setup first specifies the data construction, baseline methods, implementation parameters, and hardware environment. The following subsections then report quantitative comparisons, visual comparisons, ablation analysis, noise robustness analysis, and qualitative real-system reconstruction results.

### 4.1. Datasets, Baselines, and Implementation Details

The simulation experiments used eight scenes selected from the HCI4D light-field dataset [42]: Boxes, Cotton, Dino, Greek, Sideboard, Table, Tower, and Town. Among them, Cotton, Dino, and Sideboard were used as representative scenes for the runtime, convergence, and noise robustness analyses. Since publicly available multi-view multispectral datasets with paired ground truth are still limited, the HCI4D light-field dataset is used to construct controlled synthesized multispectral view data. For each scene, seven RGB images captured from different angular views were selected to construct a seven-channel pseudo-multispectral view-spectral tensor $X\in R^{512\times 512\times K}$, where K=7. Specifically, each pseudo-spectral channel was generated from a different angular RGB image by applying a fixed RGB-to-band linear projection. This construction preserves the multi-view geometric displacement of the HCI4D scenes and provides controlled channel-dependent variations for evaluating geometry-aware compressive reconstruction under view-dependent displacement. The channel centers were $\lambda_{k}=\left\{470,510,550,590,630,670,710\right\}$ nm. Random binary coding patterns formed C, and the coded measurement Y was generated by Equation (2).

The proposed method was compared with seven representative snapshot compressive reconstruction methods: GAP-TV [30], DeSCI [19], NLTT [33], FFD-Net [31], PnP-DIP [35], LRSDN [38], and SCI-BDVP [39]. These baselines cover optimization-based reconstruction, nonlocal low-rank modeling, tensor-prior reconstruction, plug-and-play denoising, deep-prior/self-supervised reconstruction, and recent untrained neural-network-based SCI reconstruction. All baselines were run using their official open-source implementations, and the main reconstruction parameter choices were verified to be reasonable. To make the comparison consistent, the same coded measurements, coding patterns, reconstruction size, and evaluation regions were used for all methods.

Fully supervised end-to-end and deep unfolding methods are not included as primary baselines because they usually require paired training data matched to a specific sensing matrix, channel configuration, coding pattern, and compression ratio. Such paired multi-view multispectral training data are not publicly available for the SC-MVSI setting considered in this work. Therefore, the comparison focuses on optimization-based, plug-and-play, self-supervised/deep-prior, and untrained-network methods that can be directly adapted to the proposed forward model without retraining on paired SC-MVSI data.

For this seven-channel experimental configuration, the patch size was s=6 with a 4-pixel overlap, the nonlocal search window was Ω=10×10, and the number of similar tensors was adaptively selected as M∈[6,25]. The CP rank in Equation (17) was fixed to $R_{i}=4$ as an empirical reconstruction parameter. The ADMM maximum iteration number was T=60, the stopping tolerance was $\varepsilon =10^{-4}$, and the ADMM penalty parameter in Equation (7) was $\rho =5\times 10^{-4}$. The block-offset candidate set in Equation (12) was D={−9,−8,…,9} pixels, with fixed baseline directions ${\left\{b_{k}\right\}}_{k=1}^{K}$. The initial-reference length was set to $\tau_{\mathrm{init}}=10$, which was used only to stabilize early block-offset estimation before switching to the self-guided current estimate. Cached block offsets were updated every five iterations.

The experiments were conducted on a workstation running Ubuntu 22.04 LTS with an Intel(R) Xeon(R) w7-3545 CPU; all methods were evaluated under the same settings.

### 4.2. Quantitative Comparison on Synthesized Data

Reconstruction quality was evaluated using peak signal-to-noise ratio (PSNR), structural similarity index measure (SSIM), and channel angular error (CAE). PSNR and SSIM were averaged over all reconstructed channels to measure pixel-wise fidelity and structural similarity. CAE was used to evaluate the angular consistency between the reconstructed and ground-truth channel vectors at each pixel. Let $\hat{x}\left(p\right)\in R^{K}$ and $x\left(p\right)\in R^{K}$ denote the reconstructed and ground-truth channel vectors at pixel p, respectively. The CAE over valid pixels $\Omega_{v}$ is defined as

(20) $$\mathrm{CAE}=\frac{1}{|\Omega_{v}|}\sum_{p\in \Omega_{v}}\arccos\left(\frac{\hat{x}{\left(p\right)}^{T}x\left(p\right)}{∥\hat{x}{\left(p\right)∥}_{2}{∥x\left(p\right)∥}_{2}}\right).$$

The CAE values are reported in degrees, and lower values indicate better preservation of the relative multi-channel intensity structure.

The scene-level PSNR, SSIM, and CAE values are shown in Table 1. The proposed method achieves the highest average PSNR, increasing the value from 33.06 dB for the strongest baseline DeSCI to 33.61 dB. It obtains the best PSNR on six of the eight scenes, with especially clear gains on Cotton, Dino, Greek, and Table, where view-dependent displacement and local structural details make co-located cross-channel reconstruction less reliable. These improvements indicate that the view-aligned tensor prior is effective not only for easy high-SNR scenes, but also for scenes with stronger texture, occlusion, and geometric variation.

The SSIM results show a different trend. DeSCI obtains the highest average SSIM, while the proposed method remains close to DeSCI and achieves the second-best average SSIM among the compared methods. This result suggests that stronger nonlocal smoothing in DeSCI may better preserve global structural similarity in some scenes, whereas the proposed method provides higher pixel-wise fidelity. The CAE results further show that the proposed method obtains the lowest average channel angular error. Although DeSCI gives slightly lower CAE on Sideboard and Tower, the overall average indicates that view-aligned tensor grouping tends to better preserve the relative channel structure.

### 4.3. Computational Performance and Time Consumption

In addition to reconstruction accuracy, computational cost is an important factor for evaluating iterative SCI reconstruction methods. Therefore, we further measured the execution time of the proposed method and representative CPU-based optimization baselines on three representative scenes. All methods in this comparison were executed under the same computational environment, and the reported time corresponds to the complete reconstruction process for each scene. Since the network-based baselines rely on deep-network inference or self-supervised optimization and are implemented with GPU acceleration, their runtime is strongly affected by the deep-learning framework, GPU model, memory scheduling, and implementation details. Therefore, they are not included in this CPU-based runtime comparison.

Table 2 reports the runtime comparison. The proposed method has the highest runtime among the compared CPU-based methods, with an average runtime of 3134.99 s. This additional cost mainly comes from the reference-guided view-aligned nonlocal tensor prior, which performs block-offset estimation, view-aligned patch grouping, CP low-rank approximation, and overlapping patch aggregation during ADMM iterations. Although the current implementation is computationally heavier, the results in Table 1 show that this cost is accompanied by improved reconstruction quality, especially for scenes where cross-view structural consistency is important.

**Figure 5:**
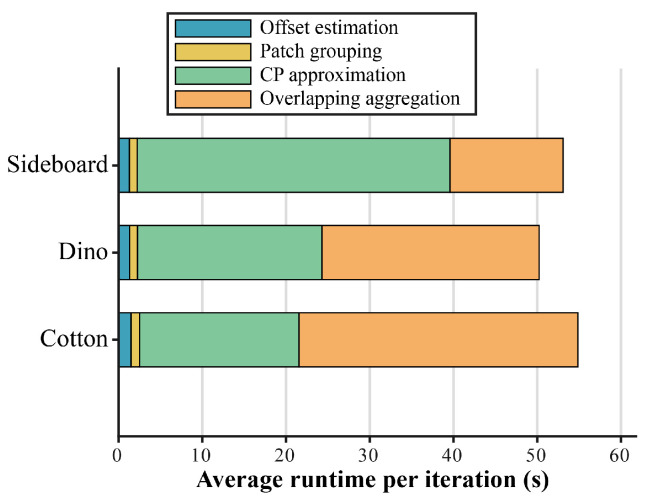
Average per-iteration runtime breakdown of the proposed method on three representative scenes. The reported modules include block-offset estimation, patch grouping, CP approximation, and overlapping aggregation.

To further analyze the computational bottleneck of the proposed method, Figure 5 shows the average per-iteration time consumption of the main modules of the proposed method. The runtime is mainly dominated by CP low-rank approximation and overlapping aggregation, while block-offset estimation and patch grouping account for a smaller proportion.

### 4.4. Visual Comparison on Synthesized Data

**Figure 6:**
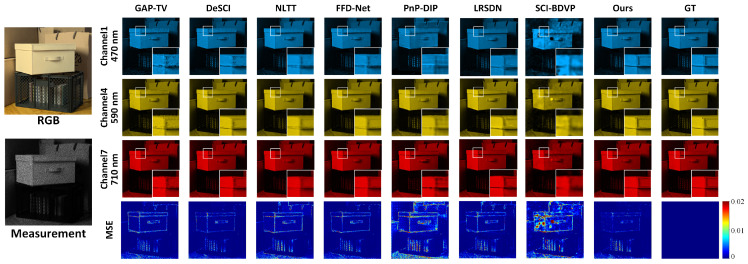
Visual comparison on the Boxes synthesized scene. The side column gives the RGB reference image and coded measurement; the main columns compare representative reconstructed channels with the ground-truth (GT) channels and include local enlargements; the bottom row shows pixel-wise mean squared error (MSE) maps relative to the GT.

**Figure 7:**
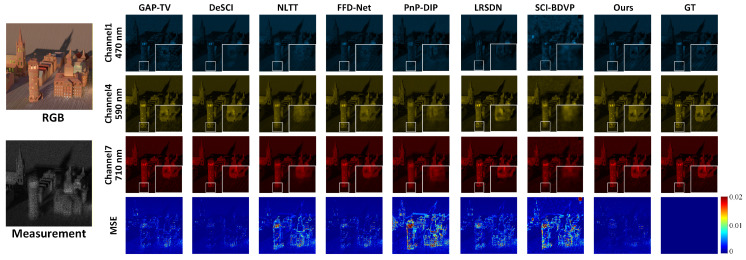
Visual comparison on the Tower synthesized scene. The side column gives the RGB reference image and coded measurement; the main columns compare representative reconstructed channels with the GT channels and include local enlargements; the bottom row shows pixel-wise MSE maps relative to the GT.

**Figure 8:**
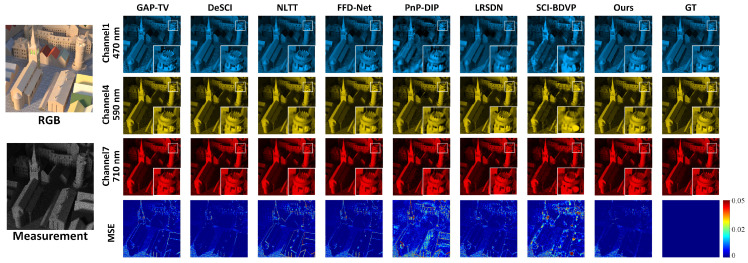
Visual comparison on the Town synthesized scene. The side column gives the RGB reference image and coded measurement; the main columns compare representative reconstructed channels with the GT channels and include local enlargements; the bottom row shows pixel-wise MSE maps relative to the GT.

Figure 6, Figure 7 and Figure 8 provide qualitative comparisons on three representative synthesized scenes, namely Boxes, Tower, and Town. For each scene, the RGB reference image and coded measurement are shown together with representative reconstructed view-spectral channels from different methods. The selected channels are displayed with wavelength-dependent colors and embedded local enlargements, while the ground-truth channel is placed next to the proposed result for direct inspection. The last row further shows pixel-wise mean squared error (MSE) distributions with respect to the ground truth. This layout highlights global reconstruction quality, local detail preservation, and the spatial distribution of reconstruction errors.

The visual results are consistent with the quantitative comparison. GAP-TV and FFD-Net tend to recover the main scene structure, but GAP-TV often contains stronger noise and local artifacts, while FFD-Net may still over-smooth fine textures because its denoising and TV priors favor locally regularized image structures. NLTT, PnP-DIP, LRSDN, and SCI-BDVP recover coarse scene structures, but their tensor- or image-specific neural priors do not explicitly correct view-dependent displacement and may still blur local details and object boundaries. For NLTT, applying low-rank tensor modeling to unaligned view-spectral channels can mix structures from different scene locations, which weakens the recovery of fine spatial content. DeSCI preserves more nonlocal structures than TV-only reconstruction, but residual blur can still appear around textured or discontinuous regions. The MSE maps provide a spatially localized view of these differences, showing that larger errors are mainly concentrated around object boundaries, textured regions, and high-contrast structures. In comparison, the proposed method generally recovers sharper local details in the enlarged regions, produces lower and more spatially confined MSE responses, and better preserves the relative channel structure, which is consistent with its lower average CAE. This behavior supports the use of block-level view alignment before nonlocal low-rank tensor grouping.

**Figure 9:**
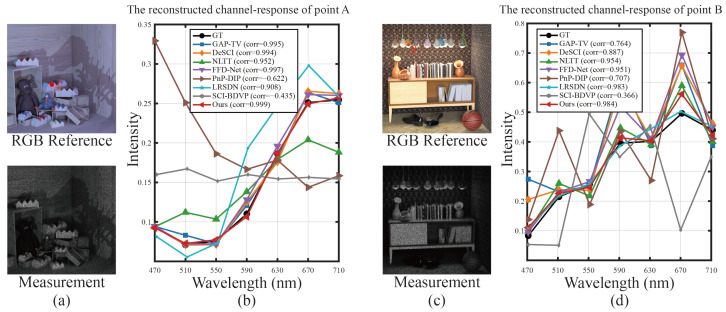
Point-wise channel-response profile comparison on the Dino and Sideboard synthesized scenes. Panels (**a**,**c**) show the RGB reference images and coded measurements with the selected points marked, and panels (**b**,**d**) compare the reconstructed channel-response profiles with the GT profiles at points A and B, respectively. Correlation coefficients in the legends are Pearson correlations with the GT profiles.

To further examine point-wise channel-vector consistency, Figure 9 compares reconstructed channel-response profiles at two selected points from the Dino and Sideboard scenes. For each selected point, the reconstructed and ground-truth (GT) profiles are sampled at the same marked image location. The Pearson correlation coefficient between each reconstructed profile and the GT profile is reported in the legend. The proposed method shows high channel–profile correlation at both selected points, indicating that the reconstructed channels preserve local channel-dependent response patterns. This result is important for SC-MVSI because reconstruction quality depends not only on single-channel image sharpness, but also on the relative intensity profile across the reconstructed channel dimension.

### 4.5. Ablation Study

Since the reference tensor is used to estimate block-level offsets and construct view-aligned tensor groups, it is not treated as an independent module separated from view alignment. To examine the contribution of the main prior components, two variants were evaluated. The full model uses the complete prior update in Section 3, including reference-guided block-offset estimation, view-aligned patch extraction, nonlocal tensor grouping, CP low-rank approximation, and overlapping aggregation. The variant without reference-guided view alignment removes this coupled branch and directly stacks cross-channel patches at identical pixel coordinates, while keeping the nonlocal grouping and CP low-rank approximation unchanged. The local tensor-only variant keeps the view-aligned local view-spectral tensor construction but removes nonlocal grouping, so the low-rank approximation is applied independently to each local tensor rather than to the fourth-order nonlocal tensor group in Equation (16).

Table 3 reports the ablation results on eight synthesized scenes. The full model achieves an average PSNR of 33.612 dB and an average CAE of 5.689 degrees. Removing reference-guided view alignment reduces the average PSNR to 31.146 dB and increases CAE to 6.886 degrees. Replacing nonlocal tensor grouping with local view-spectral tensor regularization reduces the average PSNR by 0.499 dB and increases CAE to 5.846 degrees. These results support the necessity of reference-guided view-aligned patch construction and nonlocal tensor grouping. At the same time, the local-tensor-only variant achieves a marginally higher average SSIM than the full model, with an absolute difference of only 0.0013. This small difference suggests a minor trade-off between local structural preservation and nonlocal regularization. The local-tensor-only variant processes each aligned local tensor independently, which may better preserve local contrast within small neighborhoods. By contrast, the full model exploits nonlocal redundancy through tensor grouping and CP low-rank approximation, which improves PSNR and CAE but may slightly smooth local structures when nonlocal candidates are imperfectly matched. Such imperfect matching is more likely to occur in regions with fine textures, occlusions, depth discontinuities, or large view-dependent displacement.

### 4.6. Convergence Analysis

To evaluate the empirical convergence behavior of the proposed method, we recorded the PSNR and the relative change of the reconstruction over ADMM iterations on three representative scenes. The relative change is defined as $\mathrm{relChg}:={∥X^{t}-X^{t-1}∥}_{F}^{2}/{∥X^{t-1}∥}_{F}^{2}$. Here, $X^{t}$ denotes the reconstructed tensor at the *t*-th ADMM iteration. This quantity measures the relative variation between two successive reconstructions and is used to assess the stability of the iterative process.

**Figure 10:**
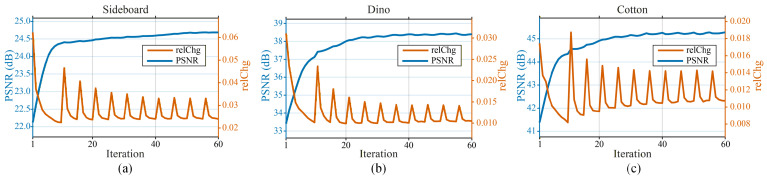
Convergence analysis of the proposed method on three representative scenes: (**a**) Sideboard, (**b**) Dino, and (**c**) Cotton. The blue curves show the PSNR across alternating direction method of multipliers (ADMM) iterations, and the orange curves show the relative change relChg between two successive reconstructions.

Figure 10 shows the convergence curves on Sideboard, Dino, and Cotton. The PSNR increases rapidly during the early iterations and then gradually stabilizes within the fixed 60-iteration setting. Meanwhile, relChg generally decreases, indicating that the reconstruction update becomes progressively smaller. The small periodic fluctuations are mainly caused by the scheduled block-offset updates, which temporarily change the view-aligned tensor grouping. These results show that the proposed method reaches stable reconstruction behavior within the iteration number used in the experiments.

### 4.7. Robustness Analysis Under Measurement Noise

To evaluate robustness to noisy measurements, zero-mean Gaussian noise was added to the 2D coded measurement while the ground-truth view-spectral tensor and coding masks were kept unchanged. Following the noisy SCI setting used in SCI-BDVP, the clean measurement $Y_{0}=A\left(X;C\right)$ was corrupted as

(21) $$Y_{\sigma }=Y_{0}+N,\;N_{ij}∼N\left(0,\sigma^{2}\right),$$

where N denotes the additive measurement noise. The noise standard deviation was set to σ∈{10,25,50}, corresponding to the measurement-domain noise levels.

Table 4 reports the reconstruction results under noisy coded measurements with σ=10, σ=25, and σ=50. As the measurement noise level increases, all methods exhibit performance degradation, indicating the increased difficulty of reconstructing view-spectral channels from corrupted coded measurements. Compared with the baselines, the proposed method generally maintains higher PSNR and favorable SSIM under different noise levels, especially under stronger measurement noise. For CAE, the proposed method achieves the best result in several cases and remains competitive in the others, although some baselines obtain lower CAE under certain noise settings. These results suggest that the reference-guided view-aligned tensor prior can improve reconstruction fidelity and structural consistency under noisy measurements, while maintaining effective cross-channel regularization in the presence of noise-induced matching disturbances.

### 4.8. Real-System Imaging Experiment

The proposed method was further evaluated using real-system measurements. Because ground-truth view-spectral channels were unavailable for the captured coded measurements, the evaluation was qualitative. The measurements were acquired with a seven-channel SC-MVSI prototype. Its imaging lens assemblies and chrome-on-quartz coding plate were custom fabricated. A primary lens and a front array of imaging lens assemblies form one central and six peripheral channel images. Each channel passes through a bandpass filter and is modulated by a corresponding random binary mask on the coding plate. Each mask contains 350×350 cells with a cell pitch of 11.6 μm. A rear array of relay lens assemblies reimages and superimposes the seven coded channel images onto a common detector area. The optical system has an effective focal length of 89.0 mm and a working F-number of 17.5. The seven filters have nominal center wavelengths of 470, 510, 550, 590, 630, 670, and 710 nm, and each filter has a nominal bandwidth of 40 nm. The multiplexed measurements were recorded with a VSS25-CL1200M monochrome CMOS camera, and each reconstructed channel has a spatial resolution of 512×512 pixels.

For the real prototype, small deviations from the idealized geometric model are unavoidable because the effective baseline lengths, channel rotations, and residual optical distortions are not perfectly identical across channels. Therefore, inter-channel geometric calibration and rectification were performed with respect to the central reference channel before reconstruction. During the prior construction for real measurements, we used a more general per-channel two-dimensional block offset, $p_{i,k}=p_{i}+\delta_{i,k}$, instead of strictly enforcing the scalar relation $p_{i,k}=p_{i}+d_{i}b_{k}$. This implementation follows the same view-aligned tensor construction principle, while allowing unequal effective baselines, different baseline directions, and residual calibration errors in the physical prototype. Nevertheless, it is still a local translational approximation and cannot fully model severe intra-block rotation, perspective deformation, occlusion, depth discontinuities, or subpixel residual displacement.

**Figure 11:**
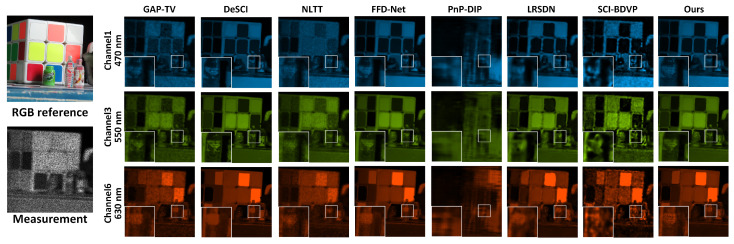
Real-system qualitative reconstruction results of the small-disparity scene. The left column shows the RGB reference image and the captured coded measurement. The remaining columns compare GAP-TV, DeSCI, NLTT, FFD-Net, PnP-DIP, LRSDN, SCI-BDVP, and the proposed method on three representative view-spectral channels with local enlarged regions.

**Figure 12:**
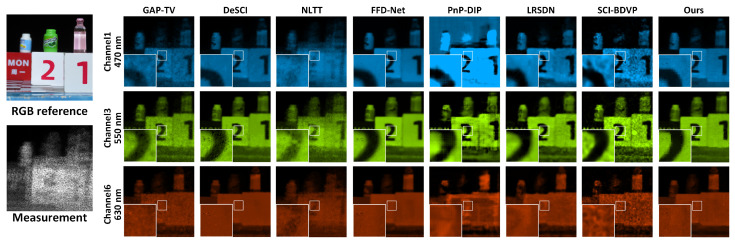
Real-system qualitative reconstruction results of the large-disparity scene. The left column shows the RGB reference image and the captured coded measurement. The remaining columns compare GAP-TV, DeSCI, NLTT, FFD-Net, PnP-DIP, LRSDN, SCI-BDVP, and the proposed method on three representative view-spectral channels with local enlarged regions.

Figure 11 and Figure 12 show two real-system captured scenes with different levels of inter-view displacement. The first scene contains relatively small inter-view disparity, so the channel misalignment is moderate. The second scene contains larger inter-view disparity, where the spatial mismatch among view-spectral channels is more evident and the reconstruction is more sensitive to inaccurate cross-channel correspondence. For each scene, the RGB reference image, the captured coded measurement, and the reconstructed results of different methods are shown for three representative channels: Channel 1 at 470 nm, Channel 3 at 550 nm, and Channel 6 at 630 nm. The representative channels are displayed with wavelength-dependent colors, and local enlarged regions are inserted to examine fine structures.

The two real scenes show different levels of geometric difficulty. In the small-disparity scene, most methods can recover the main structures, while the proposed method produces cleaner local details. In the large-disparity scene, the benefit of view-aligned tensor construction becomes more visually apparent because direct cross-channel regularization is more likely to mix structures from different scene points. This observation qualitatively supports the motivation of the proposed method, namely that tensor groups should be constructed after block-level view alignment.

## 5. Discussion

The main observation from the experiments is that channel-wise geometric inconsistency should be considered when constructing tensor priors for SC-MVSI systems. Unlike conventional coded spectral reconstruction, SC-MVSI must handle both optical compression and imperfect pixel-wise correspondence across view-spectral channels. By aligning local patches before nonlocal tensor grouping, the proposed method improves the average PSNR and reduces the average CAE, suggesting that local geometric consistency helps improve both intensity fidelity and multi-channel profile preservation. Here, CAE evaluates the angular consistency of the reconstructed channel vector at each pixel, rather than the accuracy of the estimated block offsets.

The ablation study further supports this interpretation. Removing reference-guided view alignment causes the largest degradation, indicating that direct cross-channel stacking weakens the low-rank structure of tensor groups. Replacing nonlocal grouping with local tensor regularization also reduces PSNR and increases CAE, showing that nonlocal self-similarity remains useful after view-aligned patch construction. The local tensor-only variant obtains a slightly higher average SSIM but lower PSNR and higher CAE than the full model. This suggests that local regularization may preserve small-scale structural contrast slightly better in some regions, whereas nonlocal grouping improves overall intensity fidelity and channel–profile consistency by exploiting repeated view-aligned structures.

Despite these results, this study still has some limitations. First, the quantitative evaluation is based on RGB-derived pseudo-multispectral data, which preserve multi-view geometry but cannot fully represent physically measured spectral radiance. Second, the real-system experiment is still qualitative because ground-truth view-spectral images are not available for captured coded measurements. Third, the proposed prior relies on reliable block-level offset estimation; inaccurate matching in regions with occlusion, depth discontinuities, or weak texture may reduce the benefit of view-aligned grouping. Finally, the nonlocal search, CP low-rank approximation, and overlapping aggregation introduce a higher computational cost than simpler optimization baselines. Future work will focus on calibrated real-scene benchmarks for SC-MVSI systems, more robust block matching, and faster implementations of reference-guided nonlocal tensor regularization.

## 6. Conclusions

This paper presents a view-aligned nonlocal low-rank tensor reconstruction method for SC-MVSI. The method formulates reconstruction as a coded inverse problem and solves it in an ADMM framework, where the data-consistency update enforces agreement with the detector measurement and the prior update performs reference-guided block alignment, nonlocal tensor grouping, CP low-rank approximation, and overlapping aggregation.

Experiments on eight synthesized multispectral light-field scenes show that the proposed method achieves the highest average PSNR of 33.61 dB, the lowest average CAE, and the second-highest average SSIM among the compared baselines. Ablation results confirm the contributions of reference-guided view alignment and nonlocal grouping, while real-system results provide a preliminary qualitative demonstration on captured measurements. These results demonstrate the effectiveness of applying nonlocal low-rank regularization to geometrically aligned view-spectral tensor groups in compact SC-MVSI systems.

## Figures and Tables

**Table 1:** Comparison of peak signal-to-noise ratio (PSNR), structural similarity index measure (SSIM), and channel angular error (CAE) on the synthesized dataset.

Scene	Metric	GAP-TV	DeSCI	NLTT	FFD-Net	PnP-DIP	LRSDN	SCI-BDVP	Ours
Boxes	PSNR	30.54	**33.45**	30.37	31.90	25.46	30.12	24.51	33.13
	SSIM	0.8430	**0.8971**	0.8451	0.8751	0.5832	0.7945	0.6699	0.8827
	CAE	9.89	7.29	9.45	8.22	20.21	12.17	14.45	**7.01**
Cotton	PSNR	42.77	43.69	40.46	43.78	23.53	35.77	32.14	**45.28**
	SSIM	0.9733	0.9769	0.9622	0.9776	0.7953	0.9127	0.8751	**0.9818**
	CAE	3.40	3.07	4.00	2.96	7.51	4.65	6.29	**2.77**
Dino	PSNR	33.47	37.71	32.72	35.11	26.61	31.90	27.26	**38.44**
	SSIM	0.8936	0.9538	0.8825	0.9285	0.6431	0.8285	0.7295	**0.9590**
	CAE	5.06	3.11	5.23	3.80	10.79	5.83	7.44	**2.76**
Greek	PSNR	31.17	32.81	27.71	30.81	21.72	26.89	24.06	**34.06**
	SSIM	0.8936	0.9138	0.8365	0.8951	0.5535	0.7824	0.7291	**0.9257**
	CAE	3.82	3.00	5.03	3.52	12.74	6.40	10.31	**2.84**
Sideboard	PSNR	22.25	24.07	22.36	23.26	18.55	21.85	17.98	**24.69**
	SSIM	0.6855	**0.7879**	0.6452	0.7297	0.3583	0.5873	0.4504	0.7690
	CAE	15.66	**11.70**	15.80	13.34	24.68	17.95	24.78	11.81
Table	PSNR	26.87	28.69	27.11	28.05	22.19	27.13	22.97	**29.71**
	SSIM	0.7717	**0.8256**	0.7701	0.8076	0.5187	0.7076	0.5987	0.8243
	CAE	9.19	7.09	8.38	7.48	12.97	9.72	12.84	**6.84**
Tower	PSNR	31.55	**34.69**	28.91	32.36	25.64	29.17	26.14	33.88
	SSIM	0.7994	**0.8775**	0.7526	0.8330	0.5899	0.7014	0.6494	0.8474
	CAE	7.93	**5.62**	9.48	6.71	14.64	9.96	12.79	6.26
Town	PSNR	26.89	29.37	26.95	28.04	21.55	26.61	22.21	**29.72**
	SSIM	0.8101	**0.8756**	0.7983	0.8454	0.4518	0.7303	0.6396	0.8684
	CAE	7.24	**5.22**	7.07	5.95	15.17	8.32	12.44	**5.22**
Avg.	PSNR	30.69	33.06	29.57	31.66	23.15	28.68	24.66	**33.61**
	SSIM	0.8338	**0.8885**	0.8116	0.8615	0.5617	0.7556	0.6677	0.8823
	CAE	7.78	5.76	8.06	6.50	14.84	9.38	12.67	**5.69**

Note: The best results in each row are shown in bold.

**Table 2:** Runtime comparison of the proposed method and CPU-based optimization baselines on three representative scenes.

Method	Cotton (s)	Dino (s)	Sideboard (s)	Avg. (s)
GAP-TV	1.51	1.55	1.50	1.52
DeSCI	1108.44	1125.20	1137.00	1123.55
NLTT	92.85	98.95	97.94	96.58
Ours	3178.47	2961.00	3265.51	3134.99

**Table 3:** Ablation study of the proposed reconstruction method.

Variant	Avg. PSNR (dB)	ΔPSNR	Avg. SSIM	Avg. CAE
Full model	**33.612**	**0**	0.8823	**5.689**
w/o reference-guided alignment	31.146	−2.466	0.8362	6.886
Local tensor only	33.113	−0.499	**0.8836**	5.846

Note: The best result in each column is shown in bold.

**Table 4:** Noise robustness comparison on three representative synthesized scenes. Each cell reports PSNR (dB), SSIM, and CAE (degrees) from top to bottom. The noise level σ denotes the standard deviation of the zero-mean Gaussian noise added to the coded measurement. The best result for each metric is highlighted in bold.

Method	Cotton			Dino			Sideboard		
	*σ* = 10	*σ* = 25	*σ* = 50	*σ* = 10	*σ* = 25	*σ* = 50	*σ* = 10	*σ* = 25	*σ* = 50
GAP-TV	28.08 0.7665 18.70	20.56 0.3622 35.96	14.59 0.1153 51.31	26.37 0.7610 9.34	21.07 0.4598 17.45	15.57 0.2033 29.09	21.97 0.6277 18.45	20.73 0.4792 24.16	18.01 0.3090 32.81
DeSCI	31.31 0.8864 9.65	21.52 0.4614 32.54	13.94 0.0983 52.99	28.90 0.8508 6.86	21.35 0.4922 16.87	14.86 0.1740 30.93	23.36 **0.7214** 15.30	21.03 0.5000 23.98	17.52 0.2896 34.26
NLTT	36.12 0.8663 8.96	30.90 0.6136 19.01	25.14 0.2832 33.17	31.17 0.8059 6.68	28.51 0.6056 9.44	24.54 0.3446 13.89	22.07 0.5989 17.46	21.22 0.4866 21.02	19.68 0.3584 25.63
FFD-Net	35.53 0.8548 13.10	9.51 0.2255 28.64	3.78 0.0081 24.30	32.05 0.8294 7.10	16.80 0.4901 14.74	9.38 0.1070 28.30	21.95 0.6200 17.57	12.98 0.3265 26.46	6.77 0.0724 36.91
PnP-DIP	30.08 0.7853 9.88	27.74 0.5710 19.79	22.11 0.3279 32.13	26.97 0.6613 9.18	25.22 0.5719 9.29	24.41 0.5293 **9.75**	17.83 0.3297 30.01	17.44 0.3082 26.81	16.77 0.2487 30.32
LRSDN	35.41 0.9040 **5.16**	33.59 0.8616 **7.30**	27.19 0.5078 15.97	31.60 0.8214 5.98	30.71 0.7821 **7.11**	21.97 0.2776 14.02	20.74 0.5389 19.45	20.58 0.4949 21.02	17.80 0.3209 24.64
SCI-BDVP	31.47 0.8601 7.66	29.48 0.8361 7.34	28.29 **0.7978** **9.68**	26.79 0.7261 7.48	26.67 0.7088 7.72	24.51 0.6429 12.00	18.03 0.4426 25.70	17.36 0.4056 27.68	15.67 0.3193 34.60
Ours	**39.86** **0.9531** 7.00	**35.02** **0.8852** 14.12	**29.82** 0.7121 22.89	**34.19** **0.8769** **5.12**	**31.12** **0.8153** 7.85	**27.54** **0.6976** 12.47	**24.05** 0.7106 **14.16**	**23.00** **0.6149** **16.87**	**21.44** **0.5172** **20.03**

Note: For each scene and noise level, the best result for each metric is shown in bold.

## Data Availability

The HCI4D light-field dataset used in this study is publicly available from the dataset authors. The synthesized multispectral view data, coding masks, generated measurements, reconstruction results, and evaluation scripts will be made available in a public repository upon publication. Real-system measurements are available from the corresponding author upon reasonable request.

## References

[1] Lapray P.J., Wang X., Thomas J.B., Gouton P. (2014). Multispectral Filter Arrays: Recent Advances and Practical Implementation. Sensors.

[2] Thomas J.B., Lapray P.J., Le Moan S. (2025). Trends in Snapshot Spectral Imaging: Systems, Processing, and Quality. Sensors.

[3] Han X.H., Wang J., Jiang H. (2025). Recent Advancements in Hyperspectral Image Reconstruction from a Compressive Measurement. Sensors.

[4] Hu X., Li Z., Miao L., Fang F., Jiang Z., Zhang X. (2023). Measurement Technologies of Light Field Camera: An Overview. Sensors.

[5] Imtiaz S.M., Kwon K.C., Hossain M.B., Alam M.S., Jeon S.H., Kim N. (2022). Depth Estimation for Integral Imaging Microscopy Using a 3D–2D CNN with a Weighted Median Filter. Sensors.

[6] Senussi M.F., Abdalla M., Kasem M.S., Mahmoud M., Kang H.S. (2025). U2-LFOR: A Two-Stage U2 Network for Light-Field Occlusion Removal. Mathematics.

[7] Wunsch L., Hubold M., Nestler R., Notni G. (2024). Realisation of an Application Specific Multispectral Snapshot-Imaging System Based on Multi-Aperture-Technology and Multispectral Machine Learning Loops. Sensors.

[8] Delaney B., Buntinx S., van Elst D.M.J., van Klinken A., van Veldhoven R.P.J., Fiore A. (2025). Material Sensing with Spatial and Spectral Resolution Based on an Integrated Near-Infrared Spectral Sensor and a CMOS Camera. Sensors.

[9] Hua X., Wang Y., Wang S., Zou X., Zhou Y., Li L., Yan F., Cao X., Xiao S., Tsai D.P. (2022). Ultra-Compact Snapshot Spectral Light-Field Imaging. Nat. Commun..

[10] Yuan X., Brady D.J., Katsaggelos A.K. (2021). Snapshot compressive imaging: Theory, algorithms, and applications. IEEE Signal Process. Mag..

[11] Kamal M.H., Heshmat B., Raskar R., Vandergheynst P., Wetzstein G. (2016). Tensor low-rank and sparse light field photography. Comput. Vis. Image Underst..

[12] Hao X., Zhao D., Ke J. (2025). Multiple CR Spatiotemporal Compressive Imaging System. Sensors.

[13] Shi H., Chen J., Li Y., Zhang P., Tian J. (2026). HCNet: Multi-Exposure High-Dynamic-Range Reconstruction Network for Coded Aperture Snapshot Spectral Imaging. Sensors.

[14] Yu Z., Cheng L., Ma J., Di J., Lin L., Zhan N., Zhang T., Zhong X., He X., Chen S. (2026). Spaceborne Snapshot Compressive Hyperspectral Imaging. Light Sci. Appl..

[15] Zhang X., Chen B., Zou W., Liu S., Zhang Y., Xiong R., Zhang J. (2024). Progressive content-aware coded hyperspectral snapshot compressive imaging. IEEE Trans. Circuits Syst. Video Technol..

[16] Zhang H., Wang R., Liu Y., Zhao X., Li F., Chen Y. (2025). End-to-end optimized spatial-enhanced spectral transformer for hyperspectral image reconstruction. Opt. Laser Technol..

[17] Cui Q., Park J., Ma Y., Gao L. (2021). Snapshot hyperspectral light field tomography. Optica.

[18] Zhu C., Zheng H., Yuan Y., Su L. (2023). Spectrum reconstruction of multispectral light field imager based on adaptive sparse representation. IEEE Trans. Instrum. Meas..

[19] Liu Y., Yuan X., Suo J., Brady D.J., Dai Q. (2018). Rank minimization for snapshot compressive imaging. IEEE Trans. Pattern Anal. Mach. Intell..

[20] Yuan X., Liu Y., Suo J., Durand F., Dai Q. (2021). Plug-and-play algorithms for video snapshot compressive imaging. IEEE Trans. Pattern Anal. Mach. Intell..

[21] Cao C., Li J., Wang P., Jin W., Zou R., Qi C. (2024). Hyperspectral reconstruction method based on global gradient information and local low-rank priors. Remote Sens..

[22] Cheng Z., Chen B., Lu R., Wang Z., Zhang H., Meng Z., Yuan X. (2022). Recurrent neural networks for snapshot compressive imaging. IEEE Trans. Pattern Anal. Mach. Intell..

[23] Cai Y., Lin J., Hu X., Wang H., Yuan X., Zhang Y., Timofte R., Van Gool L. Mask-guided spectral-wise transformer for efficient hyperspectral image reconstruction. Proceedings of the IEEE/CVF Conference on Computer Vision and Pattern Recognition.

[24] Zhang J., Zeng H., Chen Y., Yu D., Zhao Y.P. Improving spectral snapshot reconstruction with spectral-spatial rectification. Proceedings of the IEEE/CVF Conference on Computer Vision and Pattern Recognition.

[25] Wang J., Li K., Zhang Y., Yuan X., Tao D. (2025). *S*^2^-Transformer for Mask-Aware Hyperspectral Image Reconstruction. IEEE Trans. Pattern Anal. Mach. Intell..

[26] Meng Z., Yuan X., Jalali S. (2023). Deep unfolding for snapshot compressive imaging. Int. J. Comput. Vis..

[27] Dong Y., Gao D., Qiu T., Li Y., Yang M., Shi G. Residual degradation learning unfolding framework with mixing priors across spectral and spatial for compressive spectral imaging. Proceedings of the IEEE/CVF Conference on Computer Vision and Pattern Recognition.

[28] Cai Y., Lin J., Wang H., Yuan X., Ding H., Zhang Y., Timofte R., Gool L.V. (2022). Degradation-aware unfolding half-shuffle transformer for spectral compressive imaging. Adv. Neural Inf. Process. Syst..

[29] Gehm M.E., John R., Brady D.J., Willett R.M., Schulz T.J. (2007). Single-shot compressive spectral imaging with a dual-disperser architecture. Opt. Express.

[30] Yuan X. (2016). Generalized alternating projection based total variation minimization for compressive sensing. Proceedings of the 2016 IEEE International Conference on Image Processing (ICIP), Phoenix, AZ, USA, 25–28 September 2016.

[31] Qiu H., Wang Y., Meng D. Effective snapshot compressive-spectral imaging via deep denoising and total variation priors. Proceedings of the IEEE/CVF Conference on Computer Vision and Pattern Recognition.

[32] Chen Y., Gui X., Zeng J., Zhao X.L., He W. (2023). Combining low-rank and deep plug-and-play priors for snapshot compressive imaging. IEEE Trans. Neural Netw. Learn. Syst..

[33] Wang Y., Han Y., Wang K., Zhao X.L. (2022). Total variation regularized nonlocal low-rank tensor train for spectral compressive imaging. Signal Process..

[34] Yin X., Su L., Chen X., Liu H., Yan Q., Yuan Y. (2024). Hyperspectral image reconstruction of SD-CASSI based on nonlocal low-rank tensor prior. IEEE Trans. Geosci. Remote Sens..

[35] Meng Z., Yu Z., Xu K., Yuan X. Self-supervised Neural Networks for Spectral Snapshot Compressive Imaging. Proceedings of the 2021 IEEE/CVF International Conference on Computer Vision (ICCV).

[36] Pan Z., Zeng H., Cao J., Zhang K., Chen Y. Diffsci: Zero-shot snapshot compressive imaging via iterative spectral diffusion model. Proceedings of the IEEE/CVF Conference on Computer Vision and Pattern Recognition.

[37] Ulyanov D., Vedaldi A., Lempitsky V. Deep image prior. Proceedings of the IEEE Conference on Computer Vision and Pattern Recognition.

[38] Chen Y., Lai W., He W., Zhao X.L., Zeng J. (2024). Hyperspectral compressive snapshot reconstruction via coupled low-rank subspace representation and self-supervised deep network. IEEE Trans. Image Process..

[39] Zhao M., Chen X., Yuan X., Jalali S. (2024). Untrained neural nets for snapshot compressive imaging: Theory and algorithms. Adv. Neural Inf. Process. Syst..

[40] Kolda T.G., Bader B.W. (2009). Tensor decompositions and applications. SIAM Rev..

[41] Boyd S., Parikh N., Chu E., Peleato B., Eckstein J. (2011). Distributed optimization and statistical learning via the alternating direction method of multipliers. Found. Trends Mach. Learn..

[42] Honauer K., Johannsen O., Kondermann D., Goldluecke B. (2016). A dataset and evaluation methodology for depth estimation on 4D light fields. Proceedings of the Asian Conference on Computer Vision, Taipei, Taiwan, 20–24 November 2016.

